# Hypertrophic Scar With Contracture Over the Fourth Toe Secondary to Snake Bite Wound: To Salvage or Amputate?

**DOI:** 10.7759/cureus.9451

**Published:** 2020-07-29

**Authors:** Shir Lee Ong, Mohd Yazid Bajuri, Muhammad Haziq Abdul Suki, Fatin Nadira, Kamarul Syariza Zamri

**Affiliations:** 1 Orthopaedics and Traumatology, Universiti Kebangsaan Malaysia Medical Centre, Kuala Lumpur, MYS; 2 Orthopaedics and Traumatology, Universiti Kebangsaan Malaysia, Kuala Lumpur, MYS; 3 Orthopaedics and Traumatology, Hospital Universiti Kebangsaan Malaysia, Kuala Lumpur, MYS

**Keywords:** snake bite, hypertrophic, contracture, scar revision, realignment

## Abstract

Hypertrophic scar formation is a major clinical problem that results in both cosmetic issues and functional loss. The management of a hypertrophic scar varies according to the severity of the sequelae from the scar. We describe a method of treatment in a patient who had a history of multiple debridements due to snake bite resulting in severe contracture of the fourth toe complicated with a hypertrophic scar.

## Introduction

An injury that causes tissue loss will give rise to a repair process and eventually create scar tissue. Cutaneous wound healing is an essential physiological process that consists of a collaboration of cell strains and their products. The process can be divided into hemostasis, inflammatory, proliferation, and remodeling, which transforms a wound into granulation tissue. It requires a delicate balance between extracellular matrix protein deposition and degradation. However, if a disruption occurs, abnormalities in scarring can cause either hypertrophic or keloid scars [1]. Unnecessary wounds to any patient should be avoided to prevent pathologic scarring (keloid or hypertrophic scarring). Surgical and non-surgical management are integral in managing a keloid scar [2].

## Case presentation

**Figure 1:**
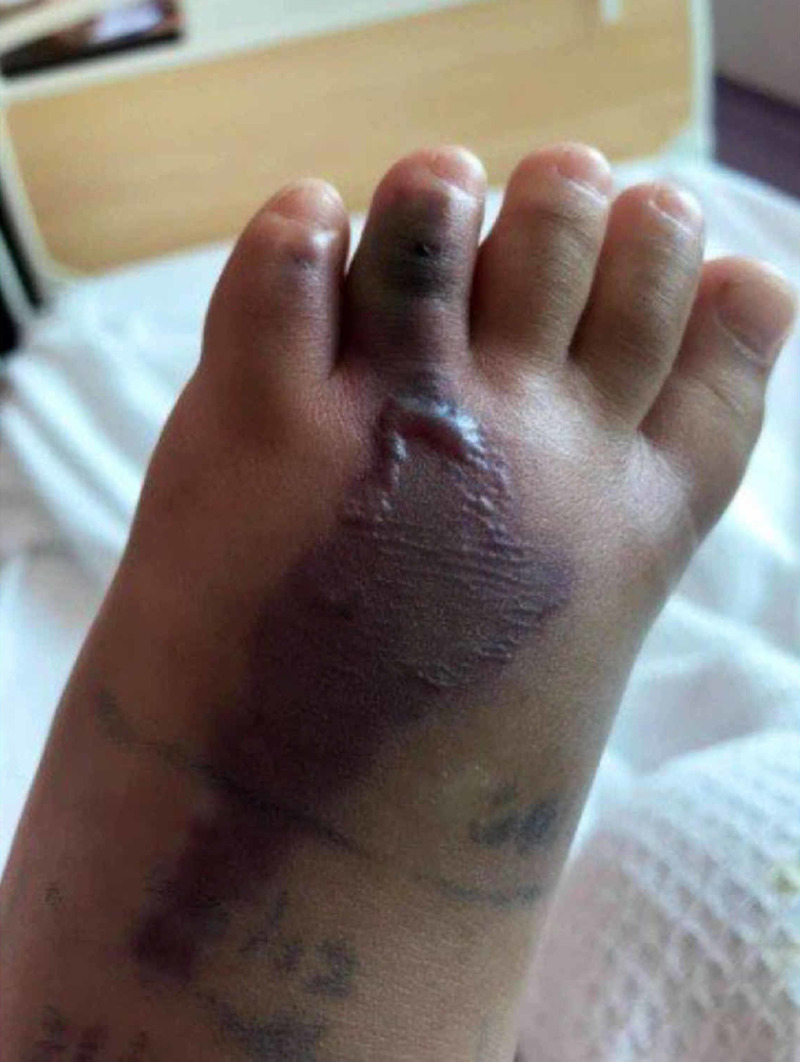
Day 3 post-injury, prior to initial treatment

**Figure 2:**
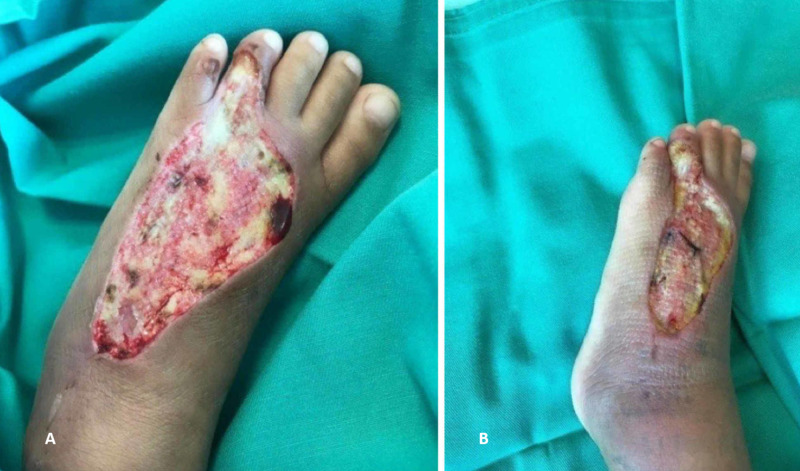
Day 1 post-surgery for fasciotomy and wound debridement. (A) Axial view showing healthy granulation tissue and contracture improve. (B) Lateral view showing healthy granulation tissue and contracture improve.

**Figure 3:**
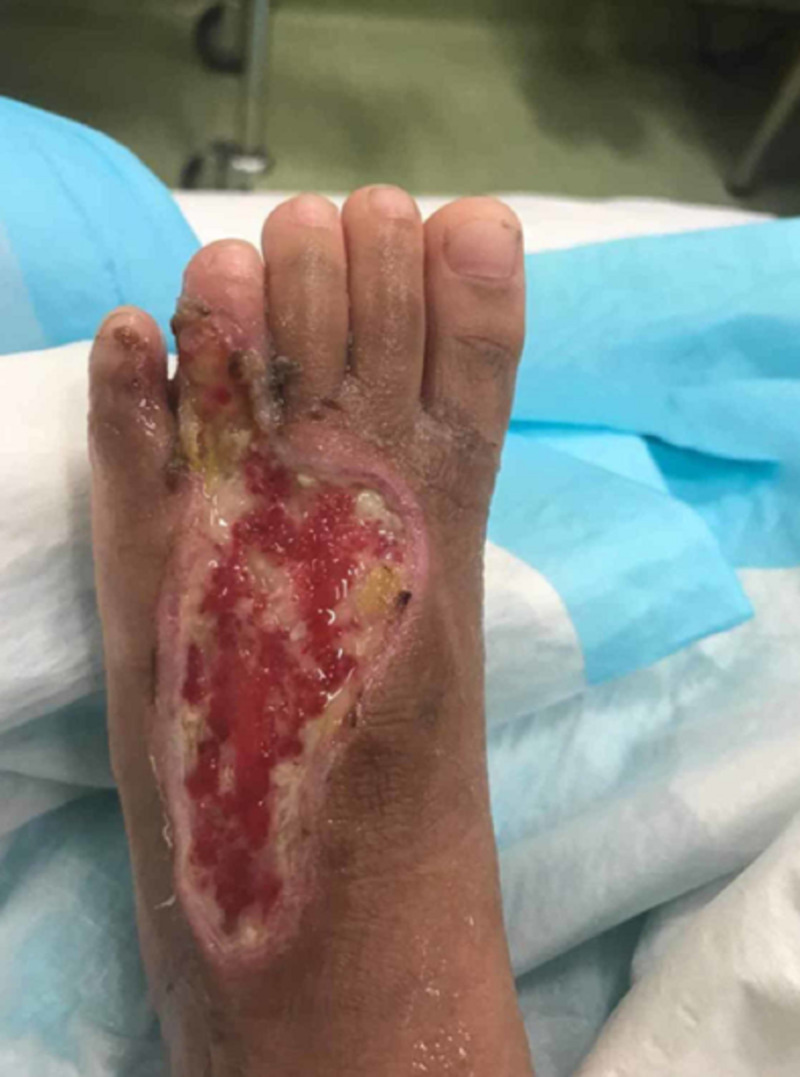
The wound after two months

**Figure 4:**
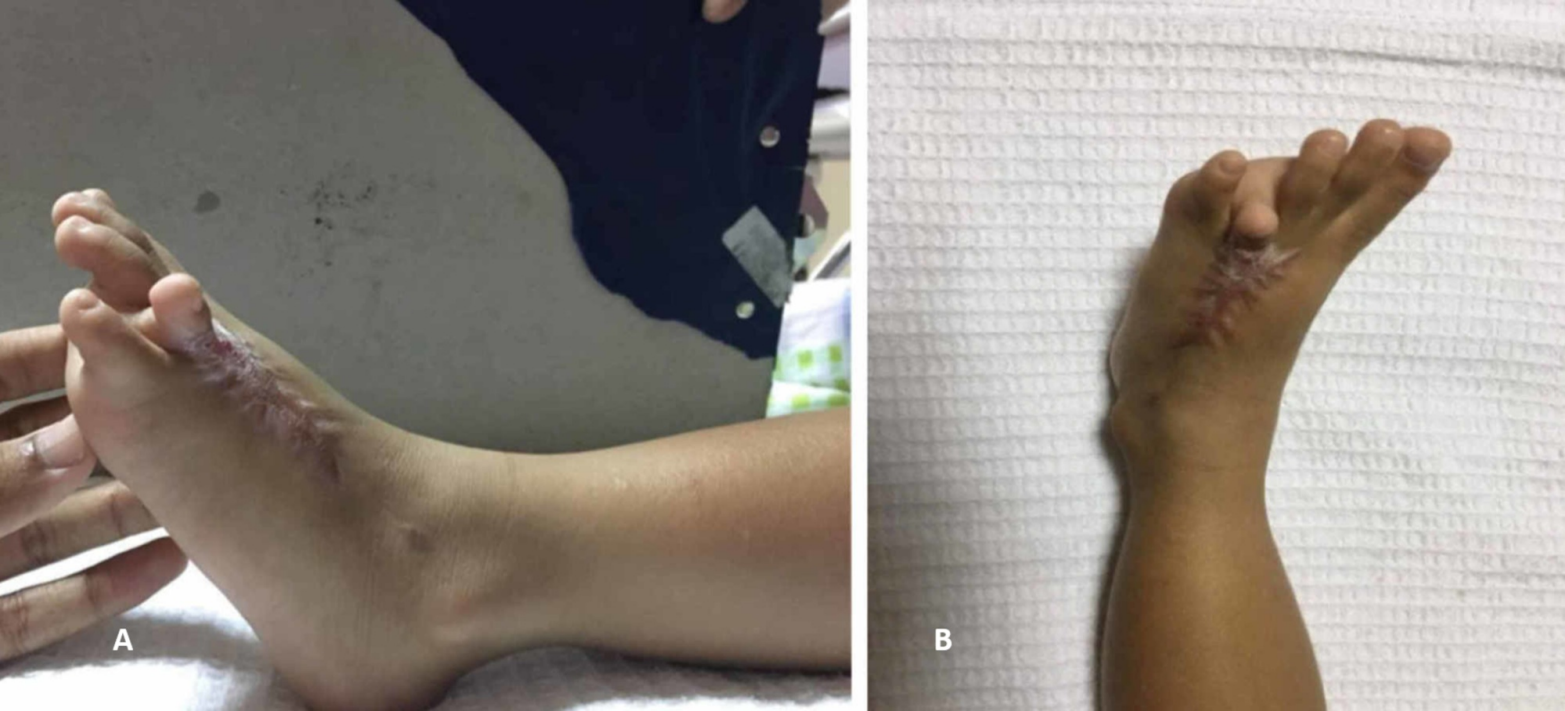
The wound after one year with contracture of the fourth toe. (A) Side view showing fourth toe in extended position. (B) Axial view showing pulp of the toe pointed upward.

A four-year-old girl presented with a history of snakebite six months prior to admission over her left foot with two puncture wounds over the dorsal surface of forefoot proximal to the fourth toe (Figure 1). Her wound was complicated with compartment syndrome and treated with fasciotomy (Figure 2). Multiple debridements were done, and the wound was allowed secondary healing (Figure 3). However, this approach resulted in a contracture of the fourth toe, affecting her footwear (Figure 4).

**Figure 5:**
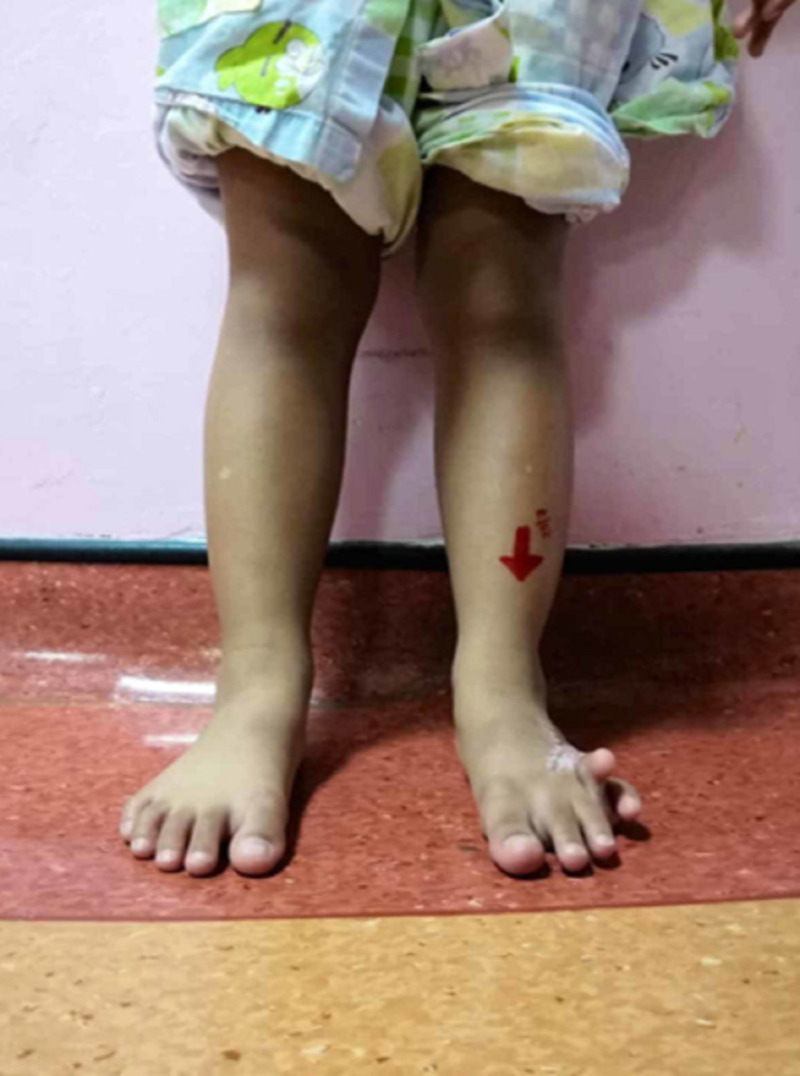
The patient’s condition prior to operation for wound revision and realignment surgery, front view

**Figure 6:**
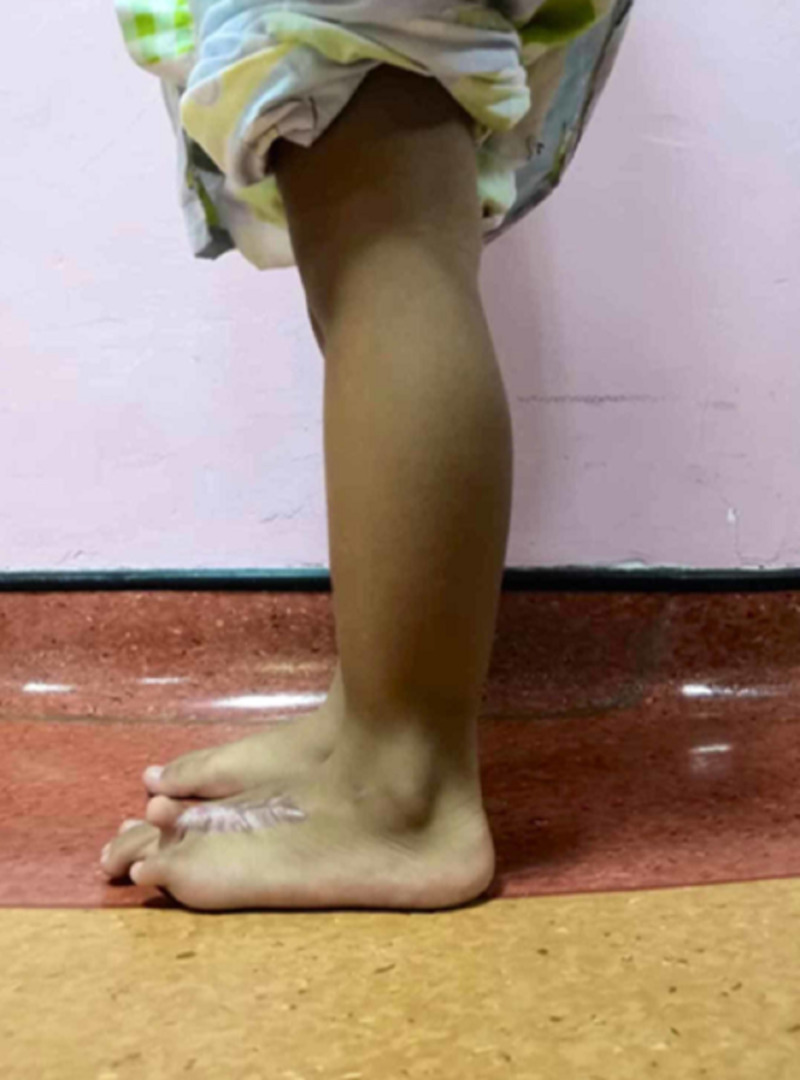
A side view of the left foot with contracture of the fourth toe

The patient was initially advised for amputation in view of the severity of the contracture (Figures 5, 6) but then presented to our center seeking a second opinion. She was advised for scar revision and realignment of the toes. 

**Figure 7:**
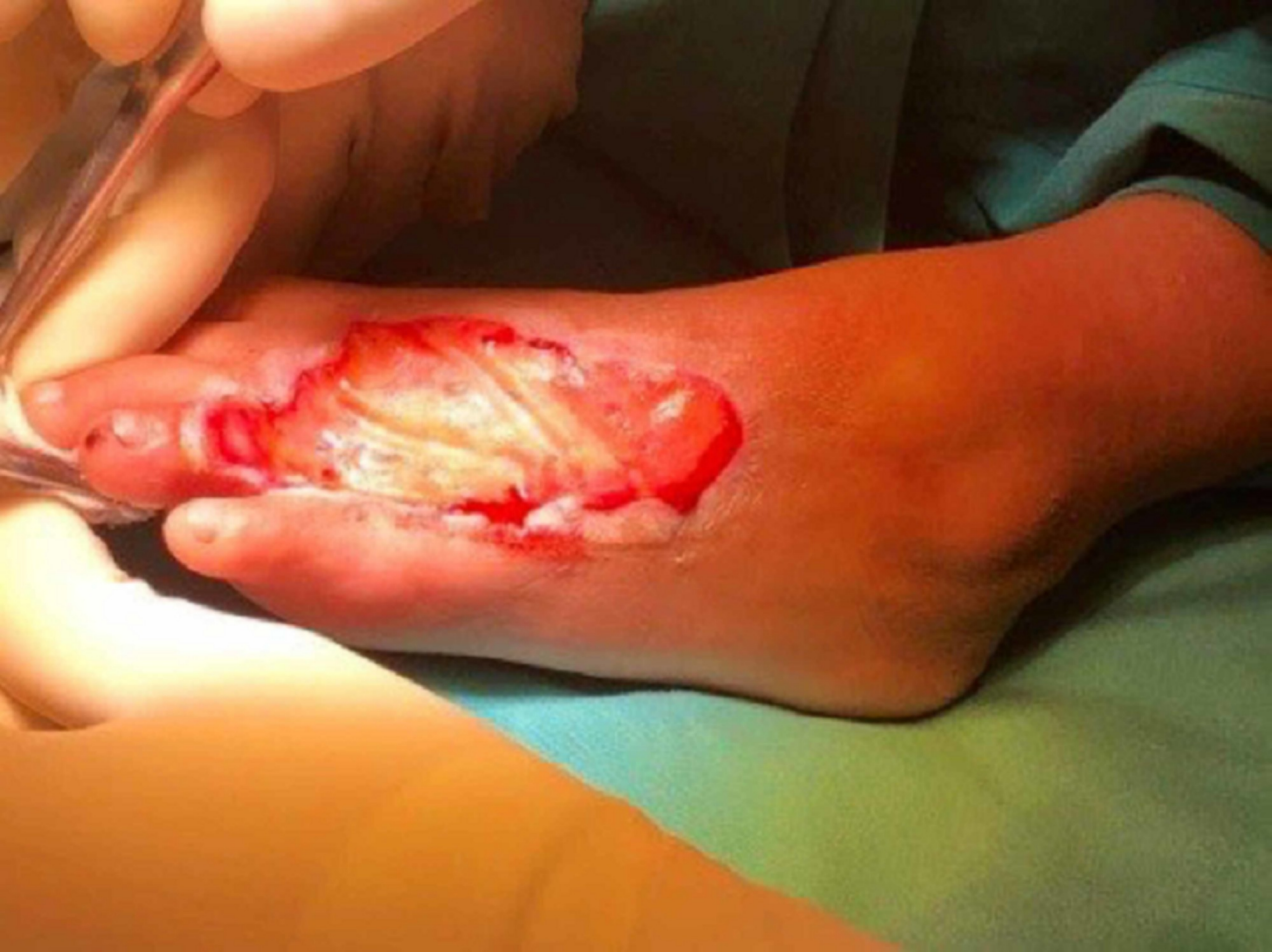
The left foot post-release of skin flap, soft tissue, muscle, tendon, and contracture

A two-stage surgery was performed. The first surgery released the skin flap, soft tissue, muscle, tendon, and contracture (Figure 7).

**Figure 8:**
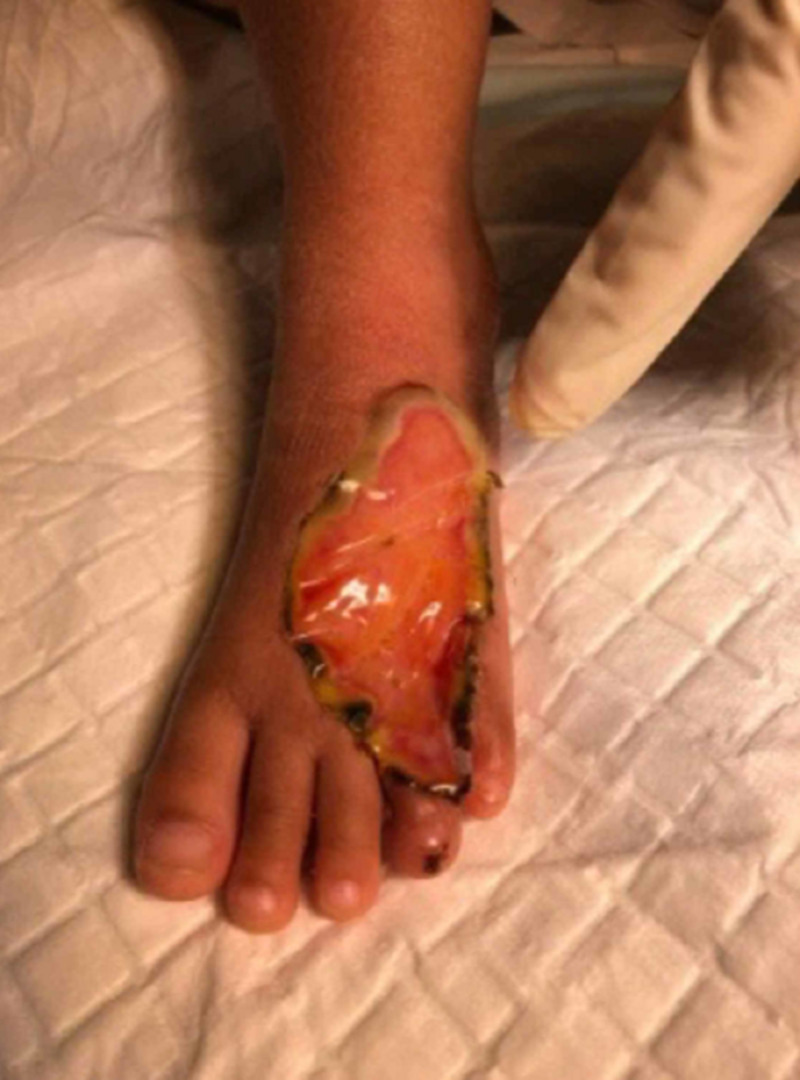
Wound covered with Integra®

Intraoperatively, we noted a hypertrophic scar over the anterolateral aspect of the dorsum of the foot from the metatarsal region to the dorsal aspect of the fourth toe and a hypertrophic capsule over the fourth metatarsophalangeal joint. We chose an elliptical incision over the dorsal aspect of the left foot for scar removal. Fibrinolysis was done until adequate soft tissue was released for fourth metatarsophalangeal joint reduction. A 0.8-mm Kirschner wire (K-wire) was inserted for joint maintenance for four weeks. The wound was covered with negative pressure dressing for three days. The patient was then posted for skin graft and Integra® (Integra LifeSciences Holdings Corporation, Plainsboro, NJ) application (Figure 8) after a satisfactory wound base was achieved (i.e., a dry wound bed).

**Figure 9:**
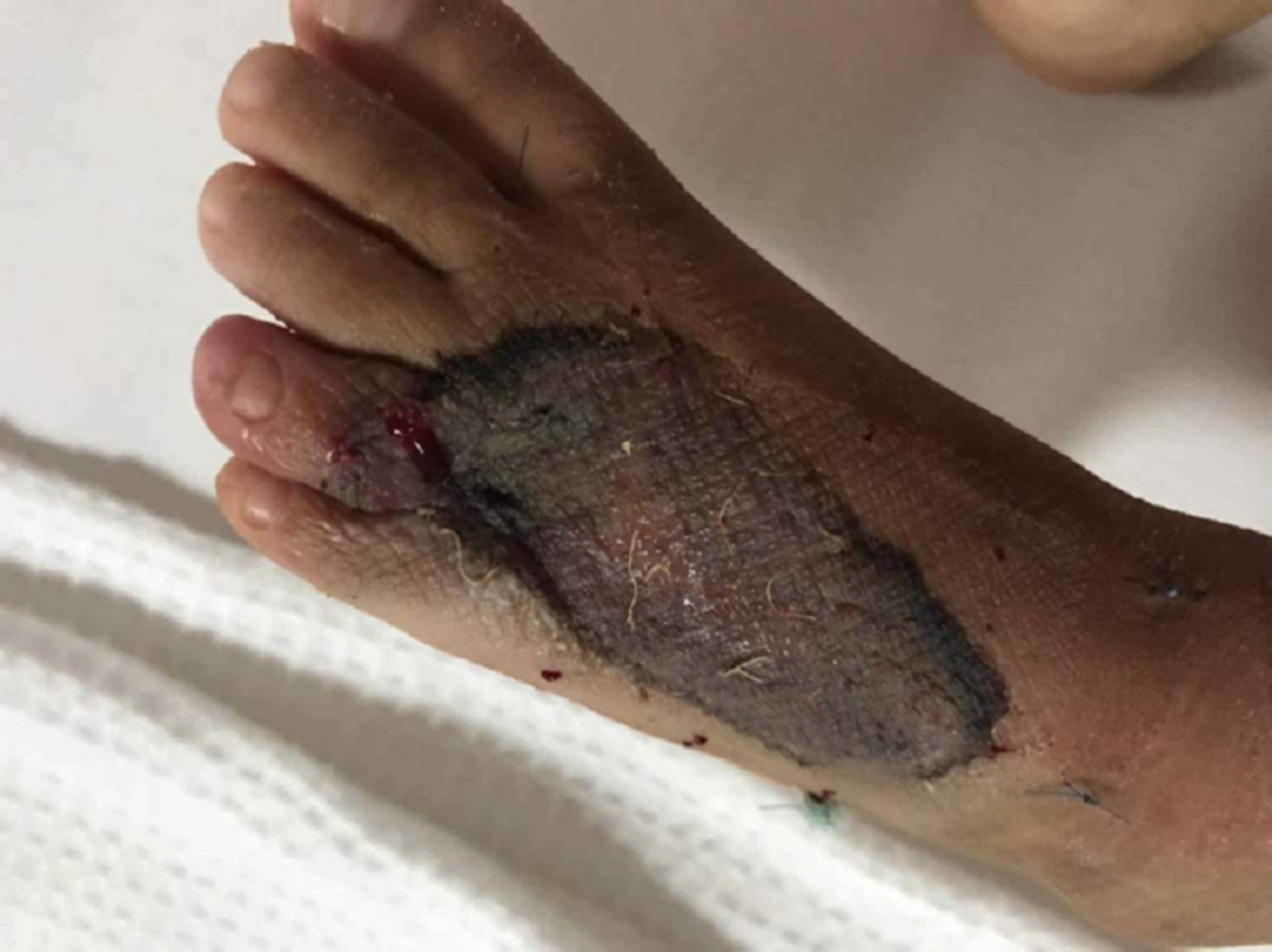
The left foot at one month

**Figure 10:**
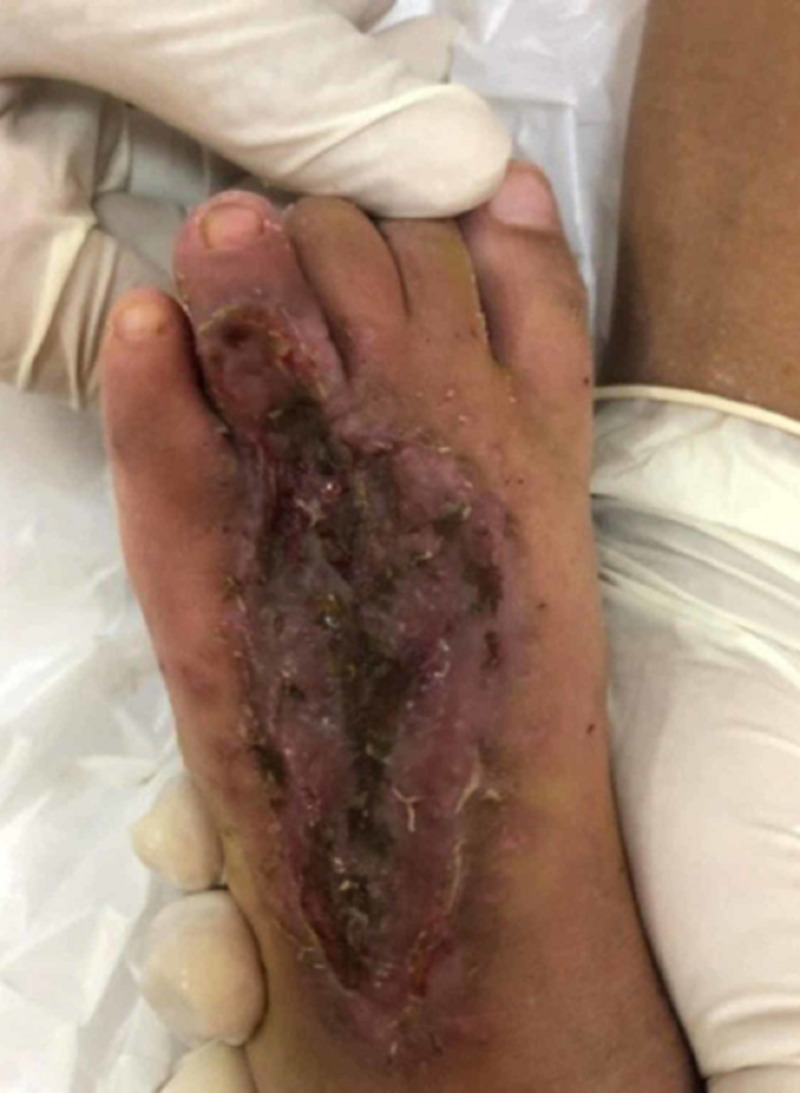
The left foot after six weeks, post-revision

Throughout the treatment, her limb was immobilized with a below-the-knee back slab, and the patient was not allowed to bear weight. She achieved a full recovery (Figures 9, 10) and complete healing with a near-normal skin texture three months after being discharged to home. At six months follow-up, the patient was able to extend and flex the toe and have normal activities. 

## Discussion

The wound healing process naturally results in a collection of fibrous tissue [3]. An ideal scar would be a fine line that traces a skin crease or fold with similar color and contour as the surrounding skin. It must not distort any adjacent structure. There is a spectrum of scars post-trauma that range from hypertrophic, atrophic, and even multinodular scar.

In the 1960s, excessive scarring was differentiated into hypertrophic and keloid scar formation [1]. Hypertrophic scars are usually caused by burns or trauma to the deep dermis that does not extend beyond the boundary of the original injury. It often occurs at an anatomical location with high tension. Delayed epithelialization beyond 10 to 14 days increases the incidence of hypertrophic scarring dramatically. A hypertrophic scar usually occurs within four to eight weeks following wound infection; closure with excess tension has a rapid growth phase of up to six months that gradually regresses over a few years. This eventually leads to a flat scar without symptoms. However, when a joint is affected, it may cause contracture. It may also cause pain, pruritus, disfigurement, and psychological issues [1-3].

Histopathologically, a hypertrophic scar can be distinguished from a keloid scar via the presence of flattening epidermis with prominent blood vessels. Scar tissue that replaced the reticular and papillary dermis also confirms the presence of a hypertrophic scar [4].

Management of hypertrophic scar can be divided according to the presence of contracture and the severity of contracture. A hypertrophic scar without contracture requires multimodal therapy, including intralesional corticosteroid injection, compression, silicone, laser, and external or internal ointment therapy [1]. A hypertrophic scar with mild contracture can be treated with complete surgical excision. However, cases with severe contracture require partial surgical contracture release with a combination of either skin graft or local flap. In the absence of satisfactory improvement, management will proceed with multimodal therapy. Otherwise, re-revision may be required.

Integra is a bilayer membrane dressing originally designed for skin replacement in burn patients. It is an artificial dermis manufactured as a synthetic bilaminate composed of a bovine collagen lattice covalently linked to chondroitin-6-sulfate (from shark cartilage) and covered with a semi-permeable polysiloxane (silicone) layer located at the outer layer to protect the deeper protein matrix [5]. Integra is produced as a type I collagen and chondroitin-6-sulfate matrix, which plays a significant role in reducing the inflammatory response to the wound by preventing fibroblast activity from accelerating beyond the control of the wound repair mechanisms, and thus prevents the formation of excessive scar tissue such as the recurrence of a hypertrophic scar. Unlike other conventional dressing such as Apligraf® (Organogenesis, Canton, MA) and Dermagraft® (Organogenesis, Canton, MA), which aids in promoting and accelerating wound healing, Integra is a regenerating dressing where regeneration of the originally injured tissue to new, healthy tissue occurs. The revascularization of Integra is dependent on host cell migration and proliferation. Integra is integrated where it is implemented, regenerating a tridimensional structure, known as neodermis, in which macrophages, fibroblasts, lymphocytes, and neovascularization are found.

Integra allows immediate closure of the wound that will restore the functional barrier of the skin. Hence, fluid loss can be reduced significantly. It also helps to prepare the wound before the final positioning of a split-thickness skin graft. It is also applicable in anatomical regions in which graft placement alone would not be able to survive, such as on tendon-exposed areas and bone.

Scar reconstruction using Integra requires two stages. First, it forms a neodermis via ingrowth of host vessels, followed by the application of a thin split-thickness skin graft when a satisfactory wound base is formed. The benefits of multistaging surgery with Integra in poorly vascularized wounds promises healing, where the direct application of a skin graft might otherwise fail [5,6]. Integra provides a pliable, durable bilaminar skin reconstruction ideal for covering the joint surface. Vacuum-assisted closure or negative pressure dressing helps a poorly vascularized wound bed take to the skin graft [7]. This therapy aids in stabilizing the local wound bed and exudate removal, and provides a barrier against bacterial invasion, promotion of neovascularization, and granulation tissue [8].

## Conclusions

The outcome of hypertrophic scarring may differ based on injury type and severity and the kind of treatment the patient receives, which constitutes a broad spectrum of therapeutic strategies. In this case, contracture over the joint area is a definitive indication for scar revision. Split skin graft alone would not likely result in an optimal outcome after revision of a contracted wound from hypertrophic scarring because the raw area post-debridement was tendon and bone. Therefore, the additional dressing was needed for scar revision.
